# Crosstalk Between Dietary Fatty Acids and MicroRNAs in the Regulation of Hepatic ApoB-Containing Lipoprotein Synthesis in Humans

**DOI:** 10.3390/ijms26104817

**Published:** 2025-05-17

**Authors:** Joanna Karbowska, Zdzislaw Kochan

**Affiliations:** 1 Department of Biochemistry, Medical University of Gdansk, 80-211 Gdansk, Poland; 2 Laboratory of Nutritional Biochemistry, Department of Clinical Nutrition, Medical University of Gdansk, 80-211 Gdansk, Poland

**Keywords:** ApoB100, VLDL, PUFA, MUFA, SFA, nutritional regulation, dyslipidemia, miR, human

## Abstract

Enhanced hepatic synthesis, assembly, and secretion of apolipoprotein B (ApoB)-containing lipoproteins elevate their plasma levels and—like their impaired clearance from the circulation—can increase cardiovascular risk. Both dietary fatty acids and microRNAs contribute to the nutrient-dependent regulation of hepatic gene expression. Together, these factors may modulate lipid and ApoB-containing lipoprotein synthesis in the liver, either exacerbating or mitigating dyslipidemia. Research continues to reveal the complexity of fatty acid–microRNA networks and highlights differences in regulating hepatic ApoB-containing lipoprotein synthesis between humans and rodents. Consequently, this review focuses on studies conducted in humans or human-derived hepatocytes.

## 1. Introduction

Apolipoprotein B (ApoB) is a large, monomeric secretory protein encoded in humans by the *APOB* gene on chromosome 2, comprising 29 exons and 28 introns [1,2,3]. The expression of the human *APOB* gene is high in both the liver and the small intestine, but each tissue employs distinct regulatory elements and mechanisms [2,3]. Hepatocytes translate the *APOB* transcript into ApoB100, a full-length protein of 4536 amino acid residues [4]. In enterocytes, RNA editing of the *APOB* transcript introduces a premature stop codon, yielding a shorter protein [5]. As a result, two ApoB isoforms circulate in plasma: ApoB100, synthesized in hepatocytes and forming the structural backbone of very low, intermediate-, and low-density lipoproteins (VLDL, IDL, LDL); and ApoB48, produced exclusively by human small-intestinal enterocytes and serving as the principal apolipoprotein of chylomicrons [6]. Because the circulating ApoB48 levels are much lower than those of ApoB100 [7], plasma ApoB mainly reflects the number of liver-derived ApoB100-containing lipoprotein particles.

Growing evidence indicates that all ApoB-containing lipoproteins of hepatic origin—VLDL, IDL, and LDL—are similarly atherogenic [7,8]. In a prospective cohort of 96,126 adults with no prior stroke or coronary or peripheral artery disease and not receiving lipid-lowering therapy, myocardial infarction (MI) risk was independently linked to the total particle count of ApoB-containing lipoproteins but not to their size or class [8]. Consistently, Mendelian randomization analyses identified ApoB as the top causal lipid-related factor for coronary artery disease (CAD), the most prevalent form of cardiovascular disease (CVD) [9,10]. These data suggest that ApoB—whose levels represent the number of atherogenic, ApoB100-containing lipoprotein particles secreted by the liver—may be the strongest determinant of CVD. Thus, reducing the hepatic synthesis of ApoB-containing lipoproteins has emerged as a promising strategy for treating and preventing CVD.

## 2. Supplementation of ω-3 Polyunsaturated Fatty Acids for Cardiovascular Risk Reduction: Evidence from Randomized Controlled Trials Shows Effects on Plasma ApoB

Earlier studies suggested cardioprotective effects of unsaturated fatty acids—particularly long-chain polyunsaturated fatty acids (PUFAs) of the ω-3 series. However, more recent randomized controlled trials (RCTs) of ω-3 PUFA supplementation to reduce CVD risk have yielded inconsistent results. Among trials that measured ApoB while evaluating ω-3 PUFA supplementation for cardiovascular outcomes, some reported a benefit from purified ethyl esters of eicosapentaenoic acid (EPA, C20:5 ω-3) [11,12], while others found ω-3 PUFA supplements ineffective in preventing cardiovascular events [13]. Most of these clinical trials administered a high dose (4 g/day) of purified EPA or an EPA–DHA mixture to statin-treated patients at elevated cardiovascular risk (Table 1). Some RCTs used mineral oil as the placebo [11,14,15], a choice that can raise low-density lipoprotein cholesterol (LDL-C) [16]. In contrast, others used corn oil [13], which primarily contains linoleic (LA, C18:2 ω-6) and oleic (OA, C18:1 ω-9) acids [17] and is considered more neutral in lipid studies than mineral oil. Differences in ω-3 PUFA formulations and potential placebo effects may have contributed to the divergent findings among these trials. Although cardiovascular outcomes have been inconsistent, these RCTs showed that ω-3 PUFA supplementation alters circulating triglyceride (TG) levels—which reflect the TG cargo of VLDL particles—and plasma ApoB levels, a marker of VLDL and LDL particle number.

## 3. Dietary Fatty Acids Affect Hepatic Synthesis and Plasma Levels of Triglycerides and ApoB: Results from Controlled-Feeding Studies

A dietary intervention study in overweight males demonstrated that consumption of a diet high in full-fat dairy products, red meat, and fast foods—providing 20% of total energy from saturated fat, i.e., enriched in saturated fatty acids (SFAs)—resulted in an increased accumulation of intrahepatic TGs [18]. Evidence from controlled-feeding studies suggests that replacing dietary saturated fat with mono- or polyunsaturated fat can induce favorable changes to the lipid profile, helping to reduce cardiovascular risk [19,20,21]. When moderately hypercholesterolemic, middle-aged men and women followed a six-week diet that increased their monounsaturated fatty acid (MUFA) intake from 7.8% to 13.7% of daily energy, thus decreasing the SFA:MUFA ratio from 1.88 to 0.53, they experienced an 11.7% reduction in low-density lipoprotein cholesterol (LDL-C) concentrations [19]. This LDL-lowering effect coincided with a 6.4% reduction in plasma ApoB concentrations. A more recent randomized, controlled-feeding crossover trial in hypercholesterolemic adults found that consuming food items prepared with 54 g/day of corn oil, which is a rich source of ω-6 PUFAs (Table 2) [17], for 21 days reduced the blood levels of total cholesterol (TC), LDL-C, VLDL-C, non-HDL-C, and ApoB and decreased LDL particle concentrations to a greater extent than did a diet with 54 g/day of extra-virgin olive oil, which contains predominantly MUFAs [22]. Another RCT showed that the daily consumption of 4 g of fish oil, which is high in long-chain ω-3 PUFAs [17], for six weeks lowered the plasma levels of TGs, VLDL TGs and VLDL ApoB in men with visceral obesity even more efficiently than did corn oil (−18% TGs, −33% VLDL TGs, and −20% VLDL ApoB for fish oil vs. corn oil) [23]. A meta-analysis of 84 controlled dietary trials summarizing the effects of different dietary fats on plasma lipids and lipoproteins found that unsaturated fatty acids, particularly PUFAs, produced the most favorable changes; in fact, replacing SFAs with PUFAs for an amount of energy of 1% reduced the TG levels by 0.010 mmol/L and the ApoB levels by 10.2 mg/dL [20]. One of the RCTs demonstrated that ω-3 PUFA-rich fish oil lowered the plasma levels of TGs primarily by decreasing hepatic ApoB production, not by altering the catabolism of ApoB-containing lipoproteins [23]. Another crossover RCT showed that substituting dietary ω-6 PUFAs for SFAs for four weeks decreased the production and number of ApoB100-containing LDL particles in dyslipidemic and insulin-resistant men [24]. These findings strongly suggest that fatty acids derived from dietary fat can regulate the hepatic synthesis of ApoB-containing lipoproteins.

## 4. Dietary Fatty Acids and MicroRNAs Regulate Hepatic *APOB* Gene Transcription

### 4.1. HNF4α Activates APOB Transcription in the Liver

Although hepatic *APOB* transcription is continuous, it is not strictly constitutive. Evidence indicates that hepatocyte nuclear factor 4α (HNF4α), which binds specific response elements in the *APOB* promoter, is likely the principal transcriptional activator of hepatic *APOB* [26]. HNF4α, encoded by the *HNF4A* gene, belongs to the human nuclear receptor superfamily of transcription factors [2,27]. The use of two distinct promoters in the *HNF4A* gene, together with alternative splicing, generates at least 12 HNF4α isoforms that can assemble into a variety of homo- and heterodimers of this nuclear receptor [28]. Although alternative splicing alters HNF4α interactions with coactivators and corepressors, modulating its transcriptional activity, all HNF4α isoforms share identical DNA-binding and ligand-binding domains [28].

### 4.2. An Essential Dietary Fatty Acid Is a Structural Cofactor of Human HNF4α

Human HNF4α preferentially and reversibly binds a single molecule of LA—an essential dietary ω-6 PUFA—within its ligand-binding domain [29]. The role of LA binding is unclear; it may stabilize the protein fold, so that with the fatty acid in the binding pocket, HNF4α spontaneously adopts a transcriptionally active conformation. Assays of HNF4α target genes showed that HNF4α functions as a ligand-independent, constitutively active transcription factor [29]. Nevertheless, its functional capacity in hepatocytes can be modulated by changes in gene expression, posttranslational modifications—primarily phosphorylation and methylation, but also acetylation, ubiquitination, and SUMOylation—and by interactions with coactivators or corepressors [30]. Moreover, HNF4α is directly targeted by several microRNAs (miRs) that downregulate its hepatic expression, and the abundance of some of these miRs appears to be influenced by fatty acids [31].

### 4.3. Human miR-34a and Fatty Acids Regulate HNF4α in the Liver

Human microRNA-34a (miR-34a) is nested within the long noncoding RNA, product of the *MIR34AHG* gene expressed in the liver and many other tissues [2,32]. The processing of the miR-34a precursor yields two products: the dominant hsa-miR-34a-5p and the less abundant hsa-miR-34a-3p [2,32].

**Figure 1:**
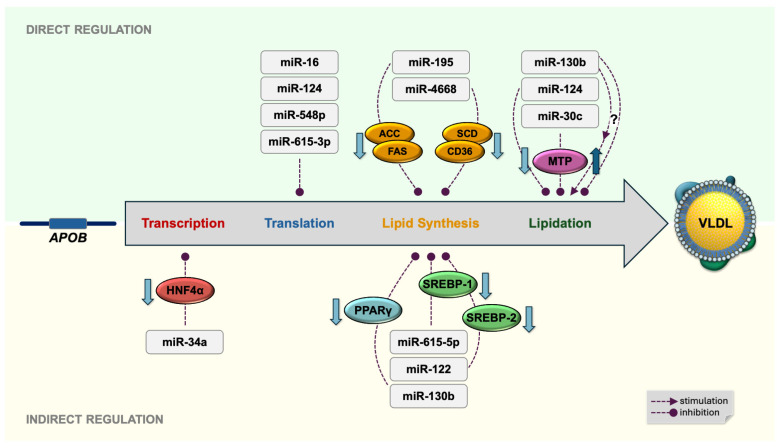
Human microRNAs that regulate hepatic ApoB-containing lipoprotein synthesis and assembly. *APOB*, gene encoding apolipoprotein B; HNF4α, hepatocyte nuclear factor 4α; PPARγ, peroxisome proliferator-activated receptor γ; SREBP-1, sterol regulatory element-binding protein 1; SREBP-2, sterol regulatory element-binding protein 2; ACC, acetyl-CoA carboxylase; FAS, fatty acid synthase; SCD, stearoyl-CoA desaturase; CD36, fatty acid translocase; MTP, microsomal triglyceride transfer protein; VLDL, very low density lipoprotein.

In the human liver, miR-34a is one of the miRs that target HNF4α mRNA. Overexpressing miR-34a in HepG2 hepatocytes significantly reduced HNF4α mRNA and protein levels [31]. Consistently, liver samples from patients with nonalcoholic steatohepatitis—who exhibited elevated hepatic TG and cholesterol levels—showed upregulated miR-34a expression along with a marked reduction in HNF4α expression [31]. In the liver, elevated miR-34a may downregulate HNF4α by degrading its mRNA. As a result, the hepatic expression of *APOB*—the HNF4α-regulated gene—is also reduced, which lowers the synthesis of ApoB-containing lipoproteins and promotes lipid accumulation in hepatocytes (Figure 1).

Treating cultured human HepG2 hepatocytes with the unsaturated oleic and linoleic fatty acids upregulated miR-34a expression [31]. In contrast, although earlier studies in HepG2 cells suggested the opposite [31], current research shows that the saturated fatty acid palmitate downregulates miR-34a expression in primary human hepatocytes and HepG2 cells [33]. A single SFA-rich meal (30 g SFA, 88% of total fat) significantly reduced the plasma miR-34a-3p levels in healthy volunteers, which was accompanied by higher plasma levels of palmitate at the expense of those of unsaturated fatty acids [34]. Although the study did not measure the dominant miR-34a-5p strand, its level likely declined as well, because both strands derive from the same precursor. In response to high levels of saturated fat, lower concentrations of miR-34a may relieve the repression of HNF4α in the liver, upregulating the hepatic expression of *APOB* and enhancing the synthesis of ApoB-containing lipoproteins.

## 5. Posttranscriptional Regulation of Hepatic ApoB Expression by Dietary Fatty Acids and MicroRNAs

**Figure 2:**
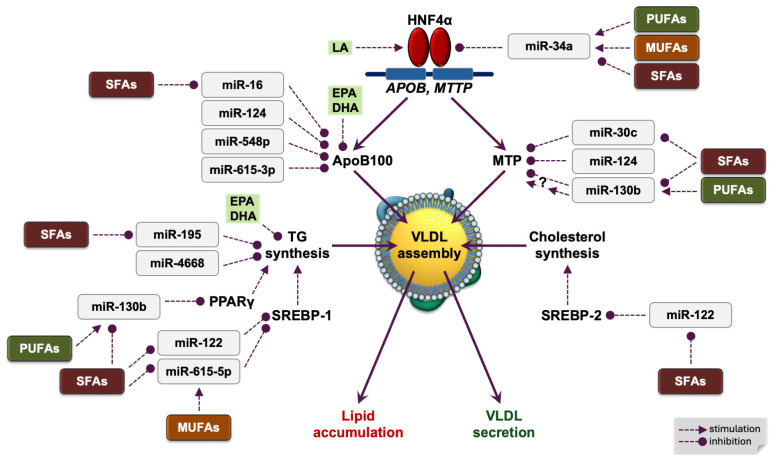
Network of fatty acids and microRNAs modulating hepatic synthesis of lipids and ApoB-containing lipoproteins. ApoB100, apolipoprotein B100; HNF4α, hepatocyte nuclear factor 4α; MTP, microsomal triglyceride transfer protein; MUFAs, monounsaturated fatty acids; PUFAs, polyunsaturated fatty acids; SFAs, saturated fatty acids; VLDL, very low density lipoprotein; TG, triglyceride; LA, linoleic acid; EPA, eicosapentaenoic acid; DHA, docosahexaenoic acid.

In the human liver, ApoB expression is also regulated by the posttranscriptional degradation of its mRNA. Evidence indicates that fatty acids can modulate this process through microRNA-mediated mechanisms (Figure 2).

### 5.1. miR-16

Two loci encode miR-16 in the human genome: *MIR16-1* (13q14.2), which resides in the intronic sequence of the *DLEU2* gene, coding for the deleted in lymphocytic leukemia 2 long noncoding RNA, and *MIR16-2* (3q25.33), located within the intronic sequence of the *SMC4* gene, coding for the structural maintenance of chromosomes 4 protein [2,32]. Both genomic regions give rise to an identical dominant hsa-miR-16-5p strand and two minor products, i.e., hsa-miR-16-1-3p and hsa-miR-16-2-3p.

Experimentally validated bioinformatics databases have recently identified the human microRNA-16-5p (miR-16-5p) as an atherosclerosis-related microRNA that targets ApoB [35]. Consistently, overexpressing miR-16 in human HepG2 and Huh-7 hepatocytes markedly reduced ApoB mRNA levels in both cell lines [35].

Research indicates that dietary SFAs may lower hepatic miR-16 expression in humans. In primary human hepatocytes, the saturated fatty acid palmitate significantly downregulated miR-16 [33]. An acute feeding study in healthy volunteers supports this finding, showing that the plasma miR-16-1-3p levels decreased after a single SFA-rich meal [34], suggesting miR-16 downregulation. By lowering the hepatic levels of miR-16, SFA-rich diets might alleviate miR-16-dependent repression of ApoB and promote the synthesis of ApoB-containing lipoproteins in the human liver. These observations align with a recent study of at-risk individuals—with metabolic syndrome, type 2 diabetes, or normal-weight obesity—which reported an adverse postprandial TG response to a Western-style diet rich in total and saturated fat [36].

### 5.2. miR-124

MicroRNA-124 (miR-124) was recently identified as a direct repressor of *APOB* in human hepatocytes [35]. Overexpression of miR-124 in HepG2 and Huh-7 cells significantly lowered ApoB mRNA levels [35].

Three loci encode miR-124 in the human genome: *MIR124-1* (8p23.1), *MIR124-2* (8q12.3), and *MIR124-3* (20q13.33) [2,32]. Processing of each primary transcript yields two mature strands, miR-124-5p and miR-124-3p [2,32].

Experimentally validated databases list miR-124-3p as a therapeutic candidate for atherosclerosis [35]. Direct evidence that dietary fatty acids regulate hepatic miR-124 expression is, however, still lacking.

### 5.3. miR-548p

Another negative regulator of ApoB expression in the human liver may be miR-548p, encoded by the *MIR548P* gene, which—like other miR-548 family members—is expressed at low levels in many human tissues [2,32]. In vitro, miR-548p promotes the posttranscriptional degradation of ApoB mRNA and reduces ApoB secretion from human primary hepatocytes and hepatoma cells [37]. The miR-548p seed interaction site in ApoB mRNA is well conserved in humans and other primates but not in rodents [37], underscoring species differences in the regulation of hepatic ApoB synthesis.

To date, no study has demonstrated that exposure to dietary fatty acids alters hepatic miR-548p expression. Until such evidence emerges, this microRNA should be regarded as a regulator of hepatic ApoB that is not yet known to be nutrient-responsive.

### 5.4. miR-615-3p

MicroRNA-615-3p (miR-615-3p) is the most recently validated direct repressor of *APOB*. In human liver-derived cells, miR-615-3p triggers the posttranscriptional degradation of ApoB mRNA, thereby lowering the levels of both intracellular and secreted ApoB100 [38].

The gene for human miR-615 resides inside the *HOXC4* gene, located on chromosome 12 (12q13.13), which encodes the homeobox protein Hox-C4 [2,32]. The primary microRNA-615 transcript undergoes processing into two mature strands: hsa-miR-615-5p and hsa-miR-615-3p [2,32]. However, experimental data indicate that only the miR-615-3p strand functions as a negative regulator of human ApoB expression in hepatocytes [38]. Moreover, miR-615-3p exclusively targets the human *APOB* transcript and does not recognize its orthologous sequences in other mammals [38].

No well-documented evidence shows whether dietary fatty acids or overall fat intake modulates miR-615-3p expression. Determining whether diet regulates this microRNA will require dedicated, targeted studies.

## 6. Fatty Acids Can Regulate the Intracellular Degradation of Hepatic ApoB Protein

ApoB100 is an essential constituent of VLDL; each ApoB-containing lipoprotein particle secreted by the liver carries one molecule of ApoB100. Quality control, achieved through co- and posttranslational degradation of the ApoB protein, ensures that only correctly folded ApoB100 molecules proceed to form VLDL. Primordial VLDL particles are assembled in the rough ER. Depending on its folding status, nascent ApoB is either translocated into the ER lumen or retained at the membrane, where proteasomal or autophagic pathways ultimately degrade misfolded proteins [39,40].

Cell culture experiments have shown that the ω-3 PUFAs EPA and DHA stimulate the posttranslational degradation of ApoB100 and decrease its secretion in VLDL particles from human HepG2 hepatocytes [39]. In contrast, the monounsaturated fatty acid oleate does not compromise nascent ApoB100 stability in these cells [39]. This lack of effect aligns with human dietary intervention studies showing that the plasma concentrations of VLDLs—both TG-rich VLDL_1_ and cholesterol-rich VLDL_2_—are not significantly altered by dietary MUFA intake [19].

## 7. Dietary Fatty Acids and MicroRNAs Modulate Hepatic Lipid Synthesis

Hepatic ApoB translation during VLDL synthesis depends on lipid availability; thus, dysregulation of intrahepatic lipid metabolism can lead to dyslipidemia. Triglycerides transported in the blood by VLDL contain fatty acids derived from dietary sources, from the circulating pool of non-esterified fatty acids, and from de novo synthesis in the liver [18]. The dietary fat composition may influence hepatic microRNA networks and modulate fatty acid synthesis in the liver.

### 7.1. Suppression of De Novo Lipogenesis by ω-3 PUFA

Studies in cultured human hepatocytes showed that treatment with the ω-3 PUFAs EPA and DHA significantly reduced the intracellular TG content [41,42]. Decreased TG accumulation resulted from the downregulation of key hepatic lipogenic enzymes: acetyl-CoA carboxylase (ACC), which catalyzes the first and rate-limiting step in the fatty acid synthesis pathway; fatty acid synthase (FAS), responsible for producing the saturated fatty acid palmitate; stearoyl-CoA desaturase (SCD), which introduces a double bond into saturated acyl-CoAs; and diacylglycerol O-acyltransferase 2 (DGAT2), the enzyme catalyzing the final step in TG synthesis [41,42]. In hepatocytes, this reduction in de novo lipogenesis is paralleled by enhanced fatty acid oxidation [41]. A human study reported similar findings, showing that in healthy men, eight-week supplementation with 4 g/day of ω-3 PUFAs (1.84 g of EPA ethyl ester and 1.52 g of DHA ethyl ester) suppressed hepatic de novo lipogenesis and increased the fatty acid oxidation rate, thus lowering the intrahepatocellular TG content [41].

### 7.2. miR-195

Emerging evidence suggests that miR-195 targets the transcripts of the lipogenic genes *ACACA* and *FASN* in the liver, downregulating their corresponding enzymes. In HepG2 hepatocytes, miR-195 overexpression markedly reduced ACC and FAS mRNA levels [43]. This pattern aligns with observations in patients with steatotic liver disease who displayed lower hepatic miR-195 and higher ACC and FAS expression [43].

Human miR-195 is encoded within the *MIR497HG* (17p13.1) host gene, part of the *MIR497-195* cluster [7,44]. During maturation, the miR-195 hairpin yields two products, hsa-miR-195-5p and hsa-miR-195-3p [45].

In a single-meal feeding study, a saturated fat-rich meal initially nearly doubled the plasma miR-195-5p levels in healthy volunteers; however, as postprandial SFAs—particularly palmitate—accumulated, miR-195-5p levels significantly decreased [34]. Palmitic acid also downregulated miR-195 in primary human hepatocytes, which coincided with a marked upregulation of *FASN* gene expression [33]. These findings indicate that SFA-induced downregulation of hepatic miR-195 might enhance de novo lipogenesis as part of the liver’s metabolic response to a high-fat diet. Such a mechanism may also underlie the adverse effect of a saturated fat-rich Western-style diet on serum TG levels in at-risk individuals [36].

### 7.3. miR-4668

Human miR-4668 is transcribed from the *MIR4668* gene on chromosome 9 (9q31.3), located within an intron of the *UGCG* gene, which encodes UDP-glucose ceramide glucosyltransferase [2,32]. The precursor hsa-miR-4668 gives rise to two mature isoforms: hsa-miR-4668-5p, the predominant species in the human liver, and the minor hsa-miR-4668-3p isoform [2,32]. Experiments in HepG2 hepatocytes have shown that miR-4668 directly suppresses the lipogenic genes *SCD* and *CD36* (encoding fatty acid translocase), lowering their mRNA abundance [43]. SCD desaturates SFAs and is necessary for TG synthesis, whereas CD36 facilitates fatty acid uptake into cells. Upregulation of miR-4668 in the liver would therefore be expected to reduce both lipid synthesis and fatty acid import.

Experimental data on whether dietary fatty acids alter hepatic miR-4668 expression are still lacking. Clarifying this point will require dedicated nutritional studies.

### 7.4. miR-130b

Human miR-130b is the product of the *MIR130B* gene, located on chromosome 22 (22q11.21) and expressed primarily in the liver [44]. The processing of the primary transcript yields two mature microRNAs, miR-130b-5p and miR-130b-3p, the latter being the predominant isoform [44,46].

**Figure 3:**
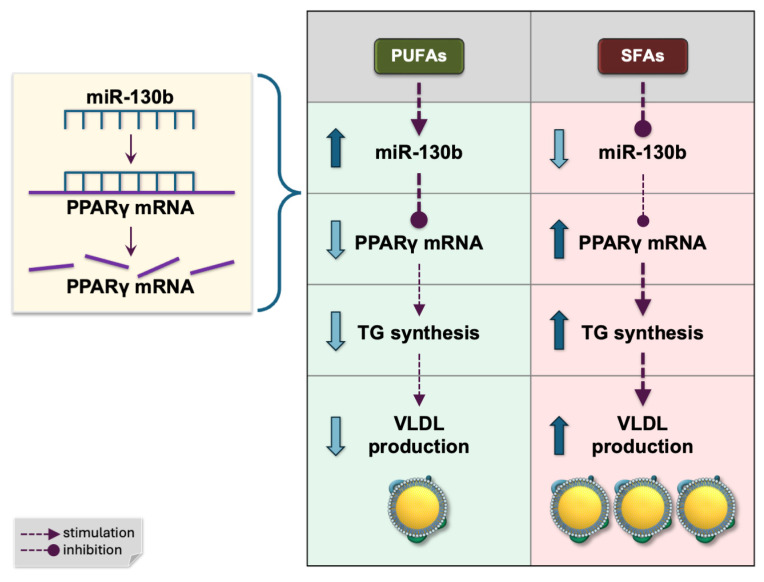
Fatty acid-dependent regulation of TG synthesis and VLDL production by hepatic miR-130b through targeting PPARγ mRNA. PUFAs, polyunsaturated fatty acids; SFAs, saturated fatty acids; PPARγ, peroxisome proliferator-activated receptor γ; TG, triglyceride; VLDL, very low density lipoprotein.

A recent study confirmed that human miR-130b-3p directly binds and represses peroxisome proliferator-activated receptor γ (PPARγ) [47]. PPARγ, a member of the nuclear receptor superfamily of transcription factors encoded by the *PPARG* (*NR1C3*) gene, acts as an intracellular fatty acid sensor that controls lipid metabolism [2,27,48,49]. In the liver, PPARγ activated by fatty acids—its natural ligands—induces the transcription of genes involved in lipogenesis [27,49]. Fatty acids may also regulate hepatic *PPARG* gene expression. The saturated fatty acid palmitate, but not the monounsaturated oleic acid, upregulated *PPARG* expression in Huh-7 hepatocytes [50]. This finding suggests that SFAs, particularly palmitate, may promote hepatic lipid synthesis by downregulating miR-130b, a negative regulator of PPARγ (Figure 3). Consistently, the hepatic expression of PPARγ and its downstream target genes is elevated in patients with nonalcoholic fatty liver disease [51].

### 7.5. miR-122

Liver-specific miR-122 is the most abundant microRNA in adult human hepatocytes [52]. In humans, miR-122 originates from a single locus within the exon of a noncoding RNA on chromosome 18 (18q21.31) [2,32]. The promoter region contains a conserved HNF4α-binding site that drives hepatic miR-122 expression [53].

Studies in HepG2 and Huh-7 hepatocytes showed that overexpressing miR-122 lowered the intracellular TG levels [54]. In the human liver, miR-122 appeared to repress FAS, a key enzyme in de novo lipogenesis, and HMG-CoA reductase (HMGCR), the rate-limiting enzyme in cholesterol synthesis [54]. Consistently, miR-122 overexpression in HepG2 and Huh-7 cells markedly reduced FAS and HMGCR at both mRNA and protein levels [54]. This downregulation is likely indirect because miR-122 also lowers sterol regulatory element-binding protein 1 (SREBP-1) and SREBP-2 mRNA and protein levels [54]. SREBP-1 is the master transcription factor for de novo fatty acid synthesis and TG formation, driving the expression of many lipogenic enzyme genes, whereas SREBP-2 regulates the expression of enzymes responsible for cholesterol synthesis [55].

Results from studies in cultured hepatic cells indicated that SFAs may decrease miR-122 levels in the human liver, as the saturated fatty acid palmitate downregulated miR-122 expression in primary human hepatocytes and HepG2 cells [33,56].

### 7.6. miR-615-5p

The mature human miR-615-5p derives from the same precursor—transcribed from an intronic miR locus within the *HOXC4* gene on chromosome 12 (12q13.13)—as that of miR-615-3p [2,32]. Processing of the primary transcript yields two mature strands: hsa-miR-615-5p and hsa-miR-615-3p [2,32].

In cultured human hepatocytes, miR-615-5p acts as a fatty-acid-responsive, negative regulator of the lipogenic transcription factor SREBP-1. Overexpressing miR-615-5p in Huh-7 cells significantly lowered SREBP-1 mRNA and protein levels, which coincided with fewer lipid droplets and a reduced intracellular TG content [57]. Treating these hepatocytes with oleic acid—at concentrations that mirrored the fasting plasma fatty acid levels in steatohepatitis patients and are known to exert a lipogenic effect—upregulated miR-615-5p expression and concomitantly downregulated SREBP-1 [57]. These findings suggest that MUFAs, particularly oleic acid, may reduce hepatic lipid synthesis by inducing miR-615-5p, which represses SREBP-1 (Figure 2). In contrast, SFAs appear to have the opposite effect: after a meal rich in saturated fat, primarily palmitic acid, miR-615-5p levels decreased in human peripheral blood mononuclear cells [58].

## 8. Lipidation of ApoB—MicroRNAs and Dietary Fatty Acids Regulating Hepatic MTP Expression

ApoB100 translation is tightly coupled to lipid availability in the liver. Because the protein contains multiple hydrophobic domains, it must interact with neutral lipids to fold correctly [4]. When the intracellular lipid supply is insufficient during VLDL assembly, hydrophobic stretches of the growing ApoB polypeptide become exposed to the aqueous environment of the ER lumen or cytosol, triggering degradation [39,40].

Cotranslational lipidation of ApoB100 is the earliest step in nascent VLDL formation. It requires several accessory proteins, the most important of which is microsomal triglyceride transfer protein (MTP) [59]. MTP, encoded by the *MTTP* gene, is an intracellular lipid transfer protein that mediates the initial incorporation of TGs, cholesterol, cholesteryl esters, and phospholipids into nascent VLDL particles in the ER, and then adds further lipids in the ER/Golgi apparatus, facilitating additional particle growth [60]. The human *MTTP* gene is highly expressed in hepatocytes and enterocytes, the two principal sites of ApoB-containing lipoprotein synthesis [2]. Hepatic *MTTP* transcription is driven by HNF4α [61] and may be indirectly downregulated by miR-34a, a microRNA that represses HNF4α.

Within the ER lumen, MTP forms a heterodimer with protein disulfide isomerase (PDI), the chaperone encoded by the *P4HB* gene. PDI catalyzes disulfide bond formation and introduces eight intramolecular disulfide bridges into mature ApoB100 [59,62]. Loss-of-function mutations in the human *MTTP* gene cause abetalipoproteinemia, i.e., the absence of ApoB-containing lipoproteins in the circulation of homozygous patients [63]. Research indicates that MTP mRNA levels in human hepatocytes can be modulated by microRNAs [37,64].

### 8.1. miR-30c

MTP is a well-established target of miR-30c in the human liver [37]. As a negative regulator of hepatic MTP expression, miR-30c binds MTP mRNA directly and promotes its posttranscriptional degradation, thereby reducing the assembly and secretion of ApoB-containing lipoproteins [64,65].

In humans, two genomic loci produce miR-30c—one hosted within the *NFYC* gene on chromosome 1 (1p34.2), which encodes nuclear transcription factor Y subunit C, and the other residing in the *LINC00472* gene on chromosome 6 (6q13), which encodes the long intergenic non-protein-coding RNA 472 [2,44]. Both loci yield an identical mature hsa-miR-30c-5p sequence—the quantitatively dominant isoform—and two minor isoforms, i.e., hsa-miR-30c-1-3p and hsa-miR-30c-2-3p [2,32].

Studies in primary human hepatocytes and HepG2 cells have shown that the long-chain saturated fatty acid palmitate downregulates miR-30c [33]. Overloading liver cells with SFAs induces ER stress [66], which may suppress miR-30c expression. Indeed, in liver samples from individuals with hepatosteatosis, miR-30c levels were significantly reduced [33]. Together, these observations suggest that dietary SFAs might decrease miR-30c levels in the human liver, thereby relieving MTP repression and enhancing VLDL synthesis. However, confirming these findings will require controlled feeding trials in humans.

### 8.2. miR-124

In the human genome, miR-124 is transcribed from three distinct loci: *MIR124-1* (8p23.1), *MIR124-2* (8q12.3), and *MIR124-3* (20q13.33) [2,32]. Processing steps convert the primary transcripts into two mature isoforms, miR-124-5p and miR-124-3p [2,32].

Recently characterized as a direct *APOB* repressor, miR-124 also targets *MTTP*; overexpression of miR-124 in HepG2 and Huh-7 hepatocytes markedly lowered both ApoB and MTP mRNA levels [35]. This dual activity of miR-124 might diminish VLDL assembly. Curated bioinformatics repositories that integrate computational predictions with experimental data highlight miR-124-3p as a promising antiatherosclerotic microRNA [35]. Whether hepatic miR-124 expression responds to dietary fatty acid intake remains unknown; controlled nutrition studies should address this question.

### 8.3. miR-130b

Human miR-130b, encoded by the *MIR130B* gene on chromosome 22 (22q11.21), is expressed primarily in the liver [44]. The processing of the primary transcript gives rise to two mature products: miR-130b-5p and the predominant isoform miR-130b-3p [44,46].

Overexpressing miR-130b-3p in HepG2 hepatocytes increased MTP mRNA abundance and VLDL secretion [67]. Because miR-130b-3p does not bind MTP mRNA directly, the authors proposed an indirect mechanism. By contrast, an eight-week normocaloric diet low in SFAs and enriched with ω-3 and ω-6 PUFAs (by adding 15 g/day each of walnuts and almonds) raised the plasma miR-130b levels and simultaneously lowered VLDL levels in healthy volunteers [68]. Experiments in primary human hepatocytes showed that the saturated fatty acid palmitate downregulates miR-130b [33], producing the opposite effect to that of PUFAs.

## 9. Final Remarks

### 9.1. Limitations

The studies described in this review have limitations that warrant careful consideration. First, human studies are often limited to microRNAs measured in plasma or serum; these circulating microRNAs do not necessarily mirror the hepatic expression, because some can also originate from other tissues. Second, many dietary trials are underpowered owing to small sample sizes and narrow participant selection. For example, the study that examined the effect of a single SFA-rich meal on the plasma miR-34a-3p, miR-16-1-3p, and miR-195-5p levels in healthy, normal-weight volunteers enrolled only women [34]. Likewise, several controlled-feeding trials that assessed how dietary fatty acids affect hepatic de novo lipogenesis and the production of ApoB-containing lipoproteins enrolled only men [18,23,24]. Because men and women differ in sex hormones, body composition, gut microbiota, and nutrient metabolism, the findings from these single-sex studies may not be fully generalizable to the opposite sex or to mixed-sex cohorts. Another study evaluated the impact of a PUFA-enriched diet on the plasma miR-130b and VLDL levels in volunteers of both sexes, all of whom had a body mass index (BMI) of 30–35 kg/m^2^ [68]. A BMI in this range indicates obesity; so, this sample choice introduced some limits to the broader applicability of the findings.

Moreover, most dietary interventions in volunteers were short—typically 3 to 8 weeks [18,19,22,23,24,68]—and many trials excluded participants with comorbidities or those taking medications [18,34,58,68]. Consequently, these studies may have failed to capture long-term benefits or harms and may have underestimated the side effects. In addition, because the study populations came from several countries on different continents, the participants likely differed in lifestyle factors, socioeconomic status, and baseline dietary patterns—such as a Mediterranean diet in Spain [58] vs. Western diets in the U.K., U.S.A., and Australia [18,19,22,23]—as well as in customary cooking methods and fats. Adherence to a Western dietary pattern is associated with higher ApoB levels, whereas individuals with lower ApoB tend to consume diets that include elements of a Mediterranean dietary pattern [69]. Finally, the diverse genetic ancestry of participants introduces variation in the frequency of single-nucleotide polymorphisms (SNPs) that affect lipid handling [70] and may therefore modulate hepatic ApoB and TG responses to dietary fatty acids.

In vitro hepatocyte models are indispensable for dissecting the mechanisms of hepatic ApoB-containing lipoprotein synthesis but introduce several limitations. First, the nonphysiologic lipid supply in culture media alters the lipidation of nascent ApoB, lowering intracellular TGs and the ApoB output [71]. Primary human hepatocytes remain the best model but display substantial donor-to-donor variability—in age, sex, genetic polymorphisms, metabolic disease, and medication history—and they rapidly dedifferentiate [72]. Long-term hepatoma lines such as HepG2 and Huh-7—owing to their low-to-moderate MTP expression and exaggerated ApoB degradation—produce fewer and smaller ApoB100-containing lipoproteins than primary human hepatocytes. In addition, their lower lipotoxic threshold, altered expression of genes implicated in lipid metabolism, defective receptor signaling, and underrepresentation of hormonal regulation [72] can distort the quantitative estimates of dietary effects on ApoB-containing lipoprotein production.

### 9.2. Species Differences in Hepatic ApoB-Containing Lipoprotein Synthesis: Humans vs. Rodent Models

The effects of dietary fatty acids and microRNAs on hepatic ApoB synthesis and the assembly of ApoB-containing lipoproteins have been studied mainly in rodents. Translating these findings to humans, however, is challenging because of inherent interspecies differences in hepatic ApoB synthesis and VLDL biology. Rodents such as mice and rats express Apobec-1—the catalytic subunit of the ApoB mRNA-editing complex, encoded by the *Apobec1* gene—not only in the small intestine but also in the liver. As a result, their hepatocytes edit ApoB mRNA and produce both ApoB48- and ApoB100-containing lipoproteins [73]. In contrast, because *APOBEC1* gene expression does not occur in the human liver, human hepatocytes cannot edit ApoB mRNA and produce only ApoB100-containing VLDL particles [74]. During the hepatic synthesis of ApoB-containing lipoproteins, ApoB—acting as a scaffold protein—determines VLDL particle size and the lipid-to-ApoB ratio. VLDL particles that contain ApoB100 are larger and more lipid-rich than those with ApoB48. Rodent VLDL particles are typically smaller than the VLDL particles produced by human hepatocytes. In addition, the basal ApoB secretion rate per unit liver mass is much higher in rodents than in humans, implying a shorter hepatic half-life for rodent ApoB-containing lipoproteins; this rapid turnover can mask modest diet-induced regulatory effects that are detectable in humans. Furthermore, unlike humans, rodents do not express cholesteryl ester transfer protein (CETP). Consequently, human VLDL carries more cholesterol and is more atherogenic than rodent VLDL, which is proportionally richer in TGs per ApoB [75].

Moreover, the mouse and rat *Apob* transcripts share only approximately 78% nucleotide identity with the human *APOB* transcript [2]. Some microRNAs interact with ApoB mRNA in a species-specific manner. For example, miR-548p and miR-615-3p bind the human *APOB* transcript but do not recognize the orthologous mouse sequences [37,38]. Because of these differences, this review focuses on studies conducted in human subjects or human hepatocytes.

### 9.3. Future Research Directions

Larger and longer randomized trials are needed to test decisively whether specific dietary fatty acids remodel hepatic microRNA networks and thereby modulate the ApoB-containing lipoprotein output in humans. Such studies should extend for at least 12 weeks because shorter interventions cannot capture durable microRNA adaptations. Replicating single-sex trials in the opposite sex and in more diverse cohorts that span a wide BMI range and include common metabolic comorbidities is also essential. MicroRNAs that proved promising in animal models [76] now merit evaluation in human in vitro systems. Because conventional hepatoma cell lines—particularly HepG2 and Huh-7—do not adequately model the complex interplay between dietary fatty acids and microRNAs that regulate the hepatic synthesis of ApoB-containing lipoproteins in humans, future research should use more physiologically relevant models. Newer hepatocyte-like in vitro systems that better replicate human lipid metabolism [77,78], along with primary human hepatocytes, are well suited to integrating these factors.

## 10. Conclusions

As the field of microRNA–nutrition interactions continues to expand, mounting evidence indicates that dietary fatty acids can regulate hepatic microRNA networks that modulate the synthesis of VLDL, the main ApoB-containing lipoproteins secreted by the human liver. Some of these microRNAs act indirectly—they target transcription factors such as HNF4α, which drives *APOB* gene expression, or proteins such as MTP, which is required for ApoB lipidation, correct protein folding, and secretion from hepatocytes. Others bind directly to ApoB mRNA, suppressing its hepatic translation. A further group of microRNAs act upstream of lipid synthesis by silencing the transcripts of lipogenic genes. By shaping these microRNA circuits, specific dietary fatty acid profiles may help lower the hepatic VLDL output and thus provide cardiovascular benefits for hyperlipidemic patients. Reformulating the fatty acid composition of foods to shift the fatty acid balance from saturated toward unsaturated species could redirect the hepatic lipid metabolism from synthesis to oxidation, reducing the production of ApoB-containing lipoproteins in the liver and complementing pharmacological therapies for atherogenic dyslipidemia.

## Figures and Tables

**Table 1:** Effects of ω-3 polyunsaturated fatty acid supplementation on plasma triglycerides and apolipoprotein B in randomized controlled trials.

Trial	Duration	Intervention	Control	Outcomes vs. Baseline		Outcomes vs. Control	
				ApoB [%]	TGs [%]	ApoB [%]	TGs [%]
MARINE [14]	12 weeks	EPA 4 g/day (n = 76)	MO (n = 75)	−3.8	–26.6	−8.5 *	−33.1 **
ANCHOR [15]	12 weeks	EPA 4 g/day + statin (n = 226)	MO + statin (n = 227)	−2.2	−17.5	−9.3 **	−21.5 **
CHERRY [12]	6–8 months	EPA 1.8 g/day + pitavastatin (n = 97)	pitavastatin (n = 96)	−9.3 **	2.9	0.6	4.4
REDUCE-IT [11]	4.9 years	EPA 4 g/day + statin (n = 4089)	MO + statin (n = 4090)	−2.5	−21.6 **	−6.7 **	−14.1 **
STRENGTH [13]	12 months	EPA + DHA 4 g/day + statin (n = 6539)	CO + statin (n = 6539)	−2.0	−19.0 **	−1.0	−18.1 **

DHA, docosahexaenoic acid; EPA, eicosapentaenoic acid; CO, corn oil; MO, mineral oil (light liquid paraffin); ApoB, apolipoprotein B; TGs, triglycerides; * *p* < 0.01; ** *p* < 0.001.

**Table 2:** Most common fatty acids in the human diet.

Fatty Acid	Type	Dietary Source
palmitic (C16:0)	SFA	palm oil, butter, beef tallow
stearic (C18:0)	SFA	butter, beef tallow, lard
palmitoleic (C16:1 ω-7)	MUFA	fish oils
oleic (C18:1 ω-9)	MUFA	olive oil, canola oil, olives
linoleic (C18:2 ω-6)	PUFA	safflower oil, corn oil, soybean oil, cottonseed oil
α-linolenic (C18:3 ω-3)	PUFA	flaxseed oil, perilla oil, canola oil, soybean oil, chia seeds, walnuts
arachidonic (C20:4 ω-6)	PUFA	meats
eicosapentaenoic (C20:5 ω-3)	PUFA	oily fish, fish oils, shellfish
docosahexaenoic (C22:6 ω-3)	PUFA	oily fish, fish oils, shellfish

SFA, saturated fatty acid; MUFA, monounsaturated fatty acid; PUFA, polyunsaturated fatty acid. Table created by the authors from data in [17,25].

## Data Availability

Not applicable.
